# Secondary neoplasms in mycosis fungoides: evaluating risk factors and phototherapy impact in a comparative study

**DOI:** 10.1007/s00277-025-06442-7

**Published:** 2025-06-17

**Authors:** Ayda Acar, Ayris Ozturk, Gunseli Ozturk, Ilgen Ertam Sagduyu, Bengu Gerceker Turk, Banu Yaman, Taner Akalın

**Affiliations:** 1 Department of Dermatology, Ege University Faculty of Medicine, 35100 Izmir, Turkey; 2 Department of Dermatology and Venereology, Usak University Training and Research Hospital, Usak, Turkey; 3 Special Office, Izmir, Turkey; 4 Department of Pathology, Ege University Faculty of Medicine, Izmir, 35100 Turkey

**Keywords:** Mycosis fungoides, Secondary neoplasm, Malignancy, Phototherapy, Beta 2-microglobulin

## Abstract

Patients with mycosis fungoides (MF) are at an increased risk of developing secondary neoplasms. We conducted a comparative analysis between MF patients with and without secondary neoplasms and non-MF patients to assess secondary neoplasm occurrence and identify potential risk factors. We aimed to evaluate the prevalence of secondary neoplasms and investigate associated risk factors in MF patients. Demographic data, treatments, phototherapy details, neoplasm occurrence and detection time, and mortality rates were collected and retrospectively analyzed for 252 MF and 278 control patients. The prevalence of secondary neoplasms was significantly higher in the MF group (13.9%) compared to the control group (3.6%) (*p* < 0.001). MF patients with neoplasms had a higher average age and older age at MF diagnosis (*p* = 0.003 and 0.016, respectively). Phototherapy type showed no significant association with neoplasm presence. Elevated initial and final beta-2 microglobulin levels were observed in MF patients with neoplasms (*p* = 0.002 and 0.005, respectively). MF patients have a higher risk of developing secondary neoplasms compared to controls. Risk increases with older age at MF diagnosis and elevated beta-2 microglobulin levels. Phototherapy was not associated with the development of secondary neoplasms in MF patients. MF patients with secondary neoplasms exhibit higher mortality rates, underscoring the importance of early detection and vigilant monitoring.

## Introduction

Mycosis fungoides (MF) is the most prevalent form of cutaneous T cell lymphoma (CTCL). Due to its diverse clinical presentations and non-specific histopathological features, particularly in the early stages, accurate diagnosis is often delayed. However, survival rates in the early stages of MF are generally favourable [1].

Patients with MF are at risk of developing secondary malignancies, especially non-melanoma skin cancer (NMSC), non-Hodgkin lymphoma (NHL) [2-4], Hodgkin lymphoma (HL) [2-5], lung cancer [2-5], bladder cancer [2-4], and malignant melanoma (MM) [2-4, 6, 7]. The development of secondary malignancies in patients with MF other than MM and NMSC is not primarily associated with the treatments received for MF. Factors such as the immunosuppressive state associated with MF, environmental carcinogen exposure, and shared genetic mutations may contribute to the development of these secondary malignancies [2, 8]. Notably, smoking is a significant risk factor, especially for lung and bladder cancer, and dermatologists are advised to encourage patients with MF to quit smoking [2]. It is also possible that the close follow-up of MF patients contributes to the detection of secondary malignancies [2, 3].

Performing total body screening, lymph node examination, comprehensive systemic questioning, age-appropriate cancer screening, and advising on smoking cessation are recommended for the dermatologist who follows up patients with MF [2].

The increased incidence of secondary malignancies in MF patients highlights the need to determine whether these patients have an elevated risk compared to the general population. This study aims to assess the risk of secondary neoplasms in MF patients and to investigate contributing factors, including demographic, clinical, laboratory characteristics and treatment modalities.

## Materials and methods

We conducted a retrospective cohort study at our phototherapy unit, analyzing data from patients with MF followed between January 1996 and July 2020, with informed consent obtained for all. The study included 252 patients with MF, who were diagnosed based on clinical and pathological correlation. Inclusion criteria required that patients have a confirmed MF diagnosis and complete clinical records. Patients with incomplete records were excluded. Collected data included gender, age, age at diagnosis of MF, MF stage, and lesion type of MF (patch, plaque, tumor, etc.), histopathological features of MF, treatment details (phototherapy type, session count, and cumulative doses), and survival status. We identified MF patients who developed secondary neoplasms, documenting neoplasm type and its timing relative to the MF diagnosis for further analysis. Both benign and malignant neoplasms were included in this analysis.

We reviewed data from 278 patients with other skin diseases (e.g., prurigo nodularis, generalized pruritus, atopic dermatitis, alopecia, vitiligo, lichen planus) treated in the same unit during the same period, excluding psoriasis, as a control group. The same criteria applied, requiring complete clinical records and phototherapy documentation. Both MF and control patients were followed regularly, with clinical data systematically recorded at each visit.

Data were organized using MS Excel, and statistical analyses were performed with Statistical Package for Social Sciences (SPSS Inc., Chicago, IL, USA, 25.0). Continuous variables, including age and phototherapy sessions, were analyzed with descriptive statistics such as means, medians, and percentages. Normality was assessed using Kolmogorov-Smirnov/Shapiro-Wilk tests. T-tests were used for normally distributed data; Mann-Whitney U and Kruskal-Wallis tests, with Dunn tests for pairwise comparisons, for non-normally distributed data. Correlations were determined using Spearman’s Rho. Categorical data were analysed with Pearson chi-square and Fisher’s exact tests. Survival analysis employed Kaplan-Meier and Cox regression models, with group comparisons via the Log Rank test. Results were assessed at a 95% confidence interval, with *p* < 0.05 deemed significant.

## Results

Our study included a total of 252 patients with MF and 278 control patients who were followed-up in our phototherapy department. The mean age in the MF group was 54.94 ± 16.9 (range: 5–92 years) and 55.17 ± 18.125 (range: 10–94 years) in the control group with no significant difference between the two groups (*p* > 0.05). Of the patients in the MF group, 132 (52.4%) were male, compared to 119 (42.8%) in the control group, indicating a statistically significant higher proportion of males in the MF group (*p* = 0.030). However, gender did not show a significant association with the development of neoplasms in either group (*p* > 0.05). Similarly, no significant relationship was found between Fitzpatrick skin type and the development of secondary neoplasms in either the MF or control groups (*p* > 0.05).

Among the 252 MF patients, 35 (13.9%) were diagnosed with 43 secondary neoplasms, compared to 10 (3.6%) with 12 neoplasms in the 278 control patients, indicating a significantly higher incidence in the MF group (*p* < 0.001). When only malignant neoplasms were considered, 32 of the 252 MF patients (12.7%) and 10 of the 278 control patients (3.6%) had at least one malignancy, and this difference remained statistically significant (*p* = 0.0002). Multiple neoplasms were observed in 7 MF patients (20%) and 2 control patients (20%). In the MF group, 9 (20.9%) neoplasms were pre-disease, while 34 (79.1%) were post-disease, with 2 patients having neoplasms both before and after MF diagnosis. In the control group, 11 (91.7%) neoplasms were post-disease, and 1 (8.3%) was pre-disease. No significant difference was found between pre- and post-disease neoplasm development in either group (*p* > 0.05). Frequencies of secondary neoplasms are presented in Table 1.

**Table 1:** Distribution of secondary neoplasms in both groups. (BCC: basal cell carcinoma, HL: hodgkin lymphoma, MM: malignant melanoma, NHL: non-Hodgkin lymphoma, SCC: squamous cell carcinoma)

Neoplasm type	MF group *n* (%)	Control group *n* (%)
**SCC**,** SCC In Situ**	4 (%9,3)	1 (%8,3)
**BCC**	10 (%23,3)	7 (%58,3)
**Pilomatricoma**	1 (%2,3)	0
**MM**	3 (%7)	0
**NHL**,** HL**,** Primary Cutaneous CD30 Lymphoproliferative Disease**	5 (%11,6)	0
**Colon adenocarcinoma**,** Tubular Adenoma with High-Grade Focal Dysplasia**	4 (%9,3)	1 (%8,3)
**Invasive ductal carcinoma**	3 (%7)	0
**Thyroid Papillary Microcarcinoma**,** Follicular Variant**,** Thyroid Follicular Neoplasm**	3 (%7)	0
**Bronchial Adenocarcinoma**,** Small Cell Lung Carcinoma**,** Lung Squamous Cell Carcinoma**	1 (%2,3)	2 (%16,7)
**Whartin’s Tumor**	2 (%4,7)	0
**Pancreatic Intraductal Papillary Mucinous Neoplasm (IPMN)**	1 (%2,3)	0
**Renal Oncocytoma**	1 (%2,3)	0
**Prostate Adenocarcinoma**	1 (%2,3)	0
**Renal Cell Carcinoma**	1 (%2,3)	0
**Gastric Epithelial Malignant Tumor**	1 (%2,3)	0
**Endometrioid Adenocarcinoma + Mucinous Adenocarcinoma**,** Uterine Cancer**	2 (%4,7)	0
**Laryngeal Neuroendocrine Carcinoma**	0	1 (%8,3)
**Total number**	43	12

The most frequently detected tumor was basal cell carcinoma (BCC) (*n* = 10), followed by hematologic malignancies (*n* = 5) and colon adenocarcinoma (*n* = 4). There was no significant difference in the frequency of malignancy types observed between the two groups (*p* > 0.05). The secondary neoplasms were classified into the groups as NMSC, MM, solid organ neoplasms, and hematologic neoplasms (Table 2). Among the detected neoplasms, solid organ tumors were the most prevalent (*n* = 20). No significant difference was detected between the two groups in terms of subgroups of malignancies (*p* > 0.05).

**Table 2:** The distribution of secondary neoplasm subgroups in both groups. (MF: mycosis fungoides, MM: malignant melanoma, NMSC: non-melanoma skin cancer)

	MF group (*n* = 252)		Control group (*n* = 278)	
Type of secondary neoplasm	Number of neoplasms *n*	Number of patients with neoplasm *n* (%)	Number of neoplasms *n*	Number of patients with neoplasm *n* (%)
**NMSC**	15	11 (4.4%)	8	7 (2.5%)
**MM**	3	3 (1.2%)	0	0
**Hematologic neoplasms**	5	5 (2%)	0	0
**Solid organ tumor**	20	17 (6.7%)	4	4 (1.4%)

The number of patients receiving phototherapy and the number of phototherapy sessions in both groups are detailed in Table 3. In the MF group, 208 patients (82.5%) received phototherapy, compared to 270 patients (97.1%) in the control group. The proportion of patients who received phototherapy in the group without secondary malignancy was significantly higher than that in the MF group with secondary neoplasm (*p* = 0.029) (Table 3). Regarding specific treatments, no significant association was observed between total NB-UVB, systemic PUVA, local PUVA, or local UVB and the presence of neoplasm in MF patients (*p* > 0.05). In the control group, patients who developed secondary neoplasms had significantly lower rates of phototherapy usage (*p* < 0.01) and total NB-UVB treatment (*p* < 0.01).

**Table 3:** The relationship between the type and number of phototherapy sessions received by patients in the MF and control groups and the presence of secondary neoplasms (NB-UVB: narrowband ultraviolet B, PUVA: psoralen + ultraviolet A, MF: mycosis fungoides)

Treatment type	Secondary neoplasm	MF group				Control group		
		Number of patients	p value	Number of sessions (Median, min-max)	p value	Number of patients	p value	Number of sessions (Median, min-max)
**No phototherapy**	Absent	33 (15.2%)	**0.029**	*	*	0	**< 0.001**	0
	Present	11 (31.4%)		*		8 (80%)		0
**NB-UVB**	Absent	122 (56.2%)	0.275	54.5 (2-360)	0.40	236 (88.1%)	**< 0.001**	26 (1-173)
	Present	16 (45.7%)		52 (15–214)		2 (20%)		20 (5–64)
**PUVA**	Absent	84 (38.7%)	0.266	69 (4-400)	0.43	34 (12.7%)	0.62	32 (1-112)
	Present	10 (28.6%)		90 (16–208)		0		0
**Local PUVA**	Absent	15 (6.9%)	0.237	51.5 (10–80)	0.27	1 (0.4%)	1.00	0
	Present	0		15 (13–34)		0		0
**Local NB-UVB**	Absent	6 (2.8%)	1.00	40 (3–72)	*	3 (1.1%)	1.00	19 (4–28)
	Present	0		*		0		*

The number of sessions for total NB-UVB and systemic PUVA was found to be significantly higher in the MF group compared to the control group (*p* < 0.001). There was no significant association between the number of sessions of total NB-UVB, systemic PUVA, or local PUVA and the development of neoplasms in MF group (*p* > 0.05). Patients in the MF group who developed secondary neoplasms received significantly more total NB-UVB sessions compared to those in the control group (*p* = 0.007). No significant association was found between cumulative doses of total NB-UVB (median dose: 53.18 ± 60.13 J (J)/cm for the MF group and 14.64 ± 49.59 J/cm^2^ for the control group), or systemic PUVA therapy (median dose: 342 ± 500.91 J/cm^2^ for the MF group and 90.36 ± 137.01 J/cm^2^ for the control group) and the occurrence of secondary neoplasms (*p* > 0.05).

Among the 44 MF patients who did not receive phototherapy, various topical and systemic treatments were administered, including topical bexarotene, acitretin, interferon, methotrexate, and electron beam therapy. Secondary neoplasms in this subgroup encompassed a wide range of malignancies, such as colon adenocarcinoma, prostate adenocarcinoma, thyroid papillary carcinoma, invasive ductal breast carcinoma, endometrioid adenocarcinoma, mucinous adenocarcinoma, and CD30-positive lymphoproliferative disorders. The number and proportion of patients who developed secondary neoplasms under each treatment modality are detailed in Table 4. Notably, electron beam therapy was the only treatment significantly associated with secondary neoplasm development (*p* = 0.032), whereas no statistically significant associations were identified with the other treatments. Furthermore, no significant relationship was observed between the type of treatment and the specific type of secondary malignancy.

**Table 4:** Treatments administered to MF patients without phototherapy and types of associated secondary neoplasms

Treatment	Number of Patients	Patients with Neoplasm (*n*, %)	Type of Secondary Neoplasms
**Acitretin**	11	2 (18.2%)	Colon adenocarcinoma (2 patients)
**Interferon**	11	4 (36.4%)	Invasive ductal breast carcinoma, Lung carcinoma, Thyroid carcinoma (2 patients)
**Topical bexarotene**	11	2 (18.2%)	Colon adenocarcinoma, Thyroid carcinoma
**Electron beam therapy**	7	4 (57.1%)	Basal cell carcinoma, Malignant melanoma, Thyroid carcinoma, CD30-positive lymphoproliferative disease
**Topical corticosteroid**	4	1 (25.0%)	Warthin tumor
**Topical retinoid**	4	1 (25.0%)	Gastric epithelial malignancy
**Photopheresis**	4	1 (25.0%)	Endometrioid adenocarcinoma, Mucinous adenocarcinoma
**Stem cell transplantation**	4	1 (25.0%)	Endometrioid Adenocarcinoma + Mucinous Adenocarcinoma, Uterine Cancer
**Radiotherapy**	4	1 (25.0%)	Squamous cell carcinoma
**Brentuximab**	4	1 (25.0%)	Prostate adenocarcinoma
**Chemotherapy**	4	1 (25.0%)	Lung carcinoma, CD30-positive lymphoproliferative disease
**Methotrexate**	4	1 (25.0%)	Colon adenocarcinoma
**Systemic bexarotene**	4	1 (25.0%)	Thyroid carcinoma
**Isotretinoin**	4	1 (25.0%)	Prostate adenocarcinoma

This table summarizes the treatment profiles of the 44 MF patients who did not receive phototherapy, along with the number and percentage of patients who developed secondary neoplasms under each treatment

In the MF group with neoplasms, the mean age was 62.71 ± 11.646 years, while in the group without neoplasms, it was 53.69 ± 17.296 years. Higher mean age in the MF group with neoplasms compared to the group without neoplasms was determined to be statistically significant (*p* = 0.003).

The mean duration of MF in the group with neoplasms was 10.34 years (median: 10 ± 5.509 years, range: 2–25 years), and 11.67 years (median: 11 ± 6.603 years, range: 1–37 years) in the group without neoplasms with no significant difference (*p* = 0.465). The mean age at MF diagnosis in the group with neoplasms was 56.23 (median: 58 ± 12.362, range: 22–78), and 44.52 (median: 45 ± 16.581, range: 4–81) in the group without neoplasms. The group with secondary neoplasms had a significantly higher mean age at MF diagnosis compared to the group without neoplasms (*p* = 0.016). Additionally, the mean age at secondary neoplasm diagnosis was 56.06 ± 10.7 years in the MF group and 72.1 ± 7.41 in the control group, with the age of onset significantly higher in the control group (*p* < 0.001). In MF patients who developed secondary neoplasms post-disease, the average time to onset was 7.37 ± 4.87 years.

The median follow-up duration for MF patients was 3 years (range: 0–21), significantly longer than the 1 year (range: 0–15) observed for the control group (*p* < 0.001). Among MF patients who developed secondary neoplasms, the median follow-up duration was 4 years (range: 0–17), while for the control group, it was 3.5 years (range: 1–8), with no significant difference noted (*p* > 0.05).

The median initial and final LDH values in the MF group with neoplasms were 199 U/L (units per liter) (range: 121–468) and 202 U/L (range: 137–3129), respectively. For the MF group without secondary neoplasms, the median initial and final LDH values were 191 U/L (range: 109–543) and 184 U/L (range: 94–748), respectively. No significant association was found between the presence of secondary neoplasms and initial or final LDH values in either group (*p* > 0.05).

The median initial and final beta-2 microglobulin values in the MF group with neoplasms were 2068 ng/mL (nanograms per milliliter) (range: 1259–3701) and 2339.5 ng/mL (range: 1448–6984), respectively. For MF patients without neoplasms, the respective values were 1815.50 ng/mL (range: 872–6050) and 1849 ng/mL (range: 988–8648). Statistically significant differences were observed, with higher initial and final beta-2 microglobulin levels in MF patients with neoplasms compared to those without (*p* = 0.002, 0.005).

The histopathological and clinical subtypes of MF did not show a significant association with the development of secondary neoplasms (Tables 5 and 6). The association between the initial and final stages of MF and the development of secondary neoplasms was not found to be significant (*p* > 0.05).

**Table 5:** Correlation between histopathologic types of MF and development of secondary neoplasm. (MF: mycosis fungoides, LCT: large cell transformation)

MF Histopathology	Presence of secondary neoplasm		*P* value
	Absent	Present	
**MF**	186 (85.7%)	28 (80%)	0.44
**Follicular MF**	16 (7.4%)	3 (8.6%)	0.73
**MF With LCT**	12 (5.5%)	4 (11.4%)	0.25
**CD8 + MF**	5 (2.3%)	1 (2.9%)	0.60
**Granulomatous MF**	1 (0.5%)	0	1.00

**Table 6:** Correlation between clinical types of MF and development of secondary neoplasm. (MF: mycosis fungoides)

	Presence of secondary neoplasm		
MF clinical type	Absent	Present	*P* value
**Classical MF**	192 (88.5%)	32 (91.4%)	0.82
**Follicular involvement**	20 (9.2%)	5 (14.3%)	0.36
**Poikilodermatous MF**	15 (6.9%)	3 (8.6%)	0.72
**Hypopigmented MF**	14 (6.5%)	0	0.23
**Erythrodermic MF**	5 (2.3%)	0	1
**Bullous MF**	1 (0.5%)	0	1
**Granulomatous MF**	1 (0.5%)	0	1

Values in parentheses indicate the percentage within the neoplasm-present (*n* = 35) or neoplasm-absent (*n* = 217) groups

No significant association was found between the treatments received other than phototherapy by MF patients (topical corticosteroid, topical bexarotene, other topical retinoids, intralesional corticosteroid, acitretin, systemic bexarotene, methotrexate, interferon, skin electron beam therapy (local or total), local radiotherapy, chemotherapy, bone marrow transplantation, photopheresis) and the development of secondary neoplasms (*p* > 0.05).

In the group of MF patients with secondary neoplasms, 8 patients (22.9%) died, while in the group without neoplasms, 22 patients (10.1%) died. The higher mortality rate in the neoplasm group was found to be significant (*p* = 0.045).

The 5- and 10-year survival rates were 88% and 81% in the MF group and 80% and 67% in the control group, respectively (Table 7), with the control group’s survival significantly lower (*p* = 0.01). In MF patients with secondary neoplasms, the 1-, 2-, 4-, 5-, and 17-year survival rates were 91%, 88%, 78%, 73%, and 36%, respectively. No deaths were observed in the control group with secondary neoplasms. Survival differences between MF and control patients with secondary neoplasms were not significant (*p* > 0.05).

**Table 7:** Survival rates of the groups

Survival time (year)	1	2	3	4	5	6	7	8	9	10	11	12	13	16	17	20
**Survival rate of MF patients (percent%)**	95	94	92	90	88		87		83	81		78	71	60	45	45
**Survival rate of control group (percent%)**	92	89	84		80	76	74	71		67	61					
**Survival rate of MF with secondary neoplasm (percent%)**	91	88		78	73										36	

In the control group, there was no significant association between primary skin disease and the development of secondary neoplasm (*p* > 0.05).

## Discussion

In this study, 252 MF patients and 278 control patients receiving phototherapy for non-MF skin conditions were assessed for secondary neoplasms. Among MF patients, 35 (13.9%) had 43 secondary neoplasms, compared to 12 neoplasms in 10 (3.6%) control patients. The incidence of secondary neoplasms was significantly higher in the MF group than in controls (*p* < 0.001). In Goyal et al.‘s study comparing 172 MF patients with 172 seborrheic dermatitis patients, the development of secondary malignancies was also found to be significantly higher in MF patients [9].

The association between MF and secondary cancers has been evaluated in many studies. These studies differ in terms of methodology, with some excluding NMSC cases, while others include secondary cancers diagnosed after a certain period (2–12 months) following the MF diagnosis. Therefore, the rates of secondary cancer observed may vary. Reported rates of MF with secondary malignancies are between 5.5% and 16.92% [2, 5, 8, 10-13]. Goyal et al.‘s meta-analysis, encompassing 10 studies from 1950 to 2019, found the rate of secondary malignancy in cutaneous T-cell lymphoma (CTCL) to range between 5.9% and 16.8% [4]. In our study, NMSC and some benign tumors such as renal oncocytoma, Warthin’s tumor, and pilomatricoma were also included. The rate of secondary neoplasms was determined to be 13.9%, which is consistent with the rates reported in the literature. To align with prior studies focusing only on malignancies, we also performed a secondary analysis excluding benign neoplasms. This analysis yielded similar findings, with a significantly higher rate of malignant neoplasms in the MF group (12.7%) compared to controls (3.6%, *p* = 0.0002).

As in the study of Goyal et al., most neoplasms in our study were solid organ tumors (46.5%); the other types included 34.9% NMSC, 11.6% hematologic malignancies, and 7% MM. In Goyal et al.’s study, 64% were solid and 36% were hematologic malignancies [8]. In Sherman et al.‘s study evaluating the risk of MM development in MF and comparing it with psoriasis patients, 47 out of 982 MF patients (4.8%) were observed to have MM development. This rate was higher than the general population and the risk of MM development in psoriasis patients. In 60% of MF patients, the diagnosis of MF was made before the diagnosis of MM [6]. In our study, we identified 3 patients (1.2%) with MM, of whom 66.6% received an MM diagnosis before MF diagnosis. Goyal et al. noted a better prognosis for patients with MF and concomitant NHL, lung cancer, and bladder cancer compared to those without MF [2]. However, we did not assess this aspect in our study.

It has been reported that MF is more commonly seen in males compared to females [14]. In our study as well, male gender was observed more frequently in the MF group (*p* = 0.03). Some studies found that the risk of developing secondary cancer in MF patients is higher in females compared to males [8, 10]. Almukhtar et al. reported an increased risk for lung cancer, CLL, HL, and NHL compared to males among female MF patients [10]. We did not find significant association between gender and the development of neoplasms in MF patients (*p* > 0.05).

Among MF patients, the group with neoplasms was found to have a significant higher average age (62.71 ± 11.646) compared to the group without neoplasms (53.69 ± 17.296) (*p* = 0.003). We observed that MF patients who developed secondary neoplasms had a significantly older age at MF diagnosis compared to those without secondary neoplasms (*p* = 0.016). In Goyal et al.‘s study, like ours, MF patients who developed malignancies had a mean age of 63, compared to 57 in the other group [8]. Additionally, Cengiz et al. noted that older age is associated with an increased risk of multiple secondary malignancies in MF patients [5].

In the MF group, among the 43 secondary neoplasms identified, 9 (20.9%) were detected prior to MF diagnosis, and 34 (79.1%) were identified post-MF diagnosis. Two patients (0.8%) had neoplasms detected both before and after MF diagnosis. In the control group, out of 12 secondary neoplasms identified, 11 (91.7%) were diagnosed after the primary skin disease, and 1 (8.3%) was diagnosed before.

In the study by Blazewicz et al., 6 cases of secondary malignancy (18.2%) were detected either before or simultaneously with the diagnosis of MF, while 27 of them (81.8%) were metachronous [11]. In Brownell et al.’s study, 112 out of 672 CTCL patients had secondary malignancies, with 82 (73.2%) diagnosed before or at the time of MF diagnosis, and 37 (26.7%) detected after MF diagnosis. In 7 patients (1%), secondary cancer was observed both before and after MF diagnosis [12]. Our study’s rates of secondary neoplasms detected before and after MF diagnosis align with those reported by Blazewicz et al. However, Brownell et al.‘s study showed a higher rate of malignancies detected before MF diagnosis. Nevertheless, our study found a similar rate of patients with both pre-existing and post-diagnosis secondary cancers as in Brownell et al.‘s study [12].

The median latency period for the development of cancer after MF diagnosis in our study is 7.5 years (0–21). The median latency period of the other studies was ranged between 2.1 and 5.4 (0–25) years [4, 8, 15].

We found no significant association between type of treatment for MF including phototherapy and development of secondary neoplasm. Among the MF patients, 11 patients (31.4%) with neoplasms and 33 patients (15.2%) without neoplasms had not received phototherapy. The group without secondary malignancy had a higher proportion of patients receiving phototherapy compared to those with neoplasms (*p* = 0.029). Sherman et al. also found no association between phototherapy and development of MM in MF patients [6]. Goyal et al. found no relationship between therapy of MF and secondary malignancy [9]. However, Scheu et al. raised concerns regarding the potential contribution of cumulative UV exposure, including both natural and phototherapy, to skin cancer development in MF patients [3]. In the subgroup of MF patients who did not undergo phototherapy, we observed a significantly higher rate of secondary neoplasms among those treated with electron beam therapy. However, no statistically significant associations were identified between other treatment modalities and secondary malignancy, and no correlation was found between specific treatment types and the histological subtype of secondary malignancies (Table 4).

Goyal et al. stated the risk of developing secondary malignancies was found to be higher in patients with tumoral MF lesions compared to those with patch and plaque lesions, as well as in patients with stage IIb and higher-stage disease compared to those with early-stage disease [9]. Similarly, Cengiz et al. reported that stage IV MF increases the risk of secondary solid tumors [5]. However, another study by Goyal et al. found no significant difference in secondary malignancy development rates between early-stage and advanced-stage MF patients [8]. We found no association between development of neoplasm and type of MF lesion and stage of MF.

In our study, a significantly higher mortality rate was observed in MF patients with neoplasms compared to those without. Similarly, Goyal et al. reported elevated mortality rates in MF patients with secondary cancer compared to those without, while Pileri et al. indicated a worse prognosis in patients with secondary malignancies [8, 15].

The retrospective design of this study may have introduced selection bias due to the exclusion of patients with incomplete records, potentially underestimating secondary neoplasm rates. The relatively short median follow-up duration (3 years) may limit the ability to fully capture long-term outcomes, and the single-center nature of the study may reduce generalizability. Additionally, causes of death could not be fully assessed due to restricted access to the electronic medical records of deceased patients.

## Conclusion

In conclusion, our study reveals an increased risk of secondary neoplasms in MF patients compared to the control group. Treatment modalities, including phototherapy, showed no association with secondary neoplasm development. These secondary neoplasms can arise both before and after the diagnosis of MF. Notably, older age at MF diagnosis and elevated initial and final beta-2 microglobulin levels emerged as risk factors for secondary neoplasm development in MF patients. Furthermore, MF patients with secondary neoplasms exhibited higher mortality rates compared to those without. These findings, alongside previous research, emphasize the importance of monitoring MF patients for the early detection and management of secondary neoplasms to improve outcomes. Additionally, special attention should be given to older patients at the time of MF diagnosis and those with elevated beta-2 microglobulin levels, as they may be at higher risk.

## Data Availability

No datasets were generated or analysed during the current study.

## Declarations

### Originality

The article is original and has not been published or submitted elsewhere.
